# The interplay of vaccination and vector control on small dengue networks

**DOI:** 10.1016/j.jtbi.2016.07.034

**Published:** 2016-10-21

**Authors:** Ross-William S. Hendron, Michael B. Bonsall

**Affiliations:** a Mathematical Ecology Research Group, Department of Zoology, University of Oxford, South Parks Road, Oxford OX1 3PS, UK; b St. Peter's College, New Inn Hall Street, Oxford OX1 2DL, UK

**Keywords:** *Aedes*, Vector, Control, Vaccination, Networks, Dengue

## Abstract

Dengue fever is a major public health issue affecting billions of people in over 100 countries across the globe. This challenge is growing as the invasive mosquito vectors, *Aedes aegypti* and *Aedes albopictus*, expand their distributions and increase their population sizes. Hence there is an increasing need to devise effective control methods that can contain dengue outbreaks. Here we construct an epidemiological model for virus transmission between vectors and hosts on a network of host populations distributed among city and town patches, and investigate disease control through vaccination and vector control using variants of the sterile insect technique (SIT). Analysis of the basic reproductive number and simulations indicate that host movement across this small network influences the severity of epidemics. Both vaccination and vector control strategies are investigated as methods of disease containment and our results indicate that these controls can be made more effective with mixed strategy solutions. We predict that reduced lethality through poor SIT methods or imperfectly efficacious vaccines will impact efforts to control disease spread. In particular, weakly efficacious vaccination strategies against multiple virus serotype diversity may be counter productive to disease control efforts. Even so, failings of one method may be mitigated by supplementing it with an alternative control strategy. Generally, our network approach encourages decision making to consider connected populations, to emphasise that successful control methods must effectively suppress dengue epidemics at this landscape scale.

## Highlights

• Genetic variants of SIT provide alternative control solutions for dengue spread.
• Vaccination and vector control can be combined to control dengue on small networks.
• Reduced vector control or imperfectly efficacious vaccines will impact dengue spread.
• Control method failings can be mitigated by using alternative control strategy.

## 1 Introduction

Dengue fever is a significant disease that is estimated to affect two and a half billion people (Whitehorn and Farrar, 2010). Weak control programmes, large scale movement of asymptomatic carriers and expanding populations of *Aedes aegypti* present a mounting challenge that is spreading across the tropics (WHO, 2015). This public health issue has rapidly escalated; before 1970 only nine countries recorded severe dengue cases, now more than 125 countries are classified as dengue endemic, with almost 400 million infections globally every year (Murray et al., 2013).

Once human hosts are bitten by a dengue-carrying vector, viral infection can lead to fever symptoms which persist for approximately one week. Following this an immune response is mounted to clear the infection, at which point hosts enter a period of cross-immunity to all serotypes (Gibbons and Vaughn, 2002). It is worth noting that an issue with dengue control is that three-quarters of annual dengue infections are asymptomatic, but hosts continue to infect mosquitoes whilst going about their daily routines (Duong et al., 2015). A more severe version of the disease manifests as dengue haemorrhagic fever. This particular quirk of dengue virus infections is the result of an immunological phenomenon called antibody dependent enhancement (Gubler, 1998). This occurs when antibodies that have been produced in response to one serotype actually facilitate rapid cell entry for a different serotype of the virus, leading to faster host body invasion and more severe secondary infections.

The dengue virus is a member of the flavivirus family, with other notable members causing West Nile disease, Zika, yellow fever and chikungunya (Amaku et al., 2011). Multiple serotypes of dengue virus exist, with the latest classified, DENV-5, having only recently been discovered (Normile, 2013). Due to antibody-dependent enhancement, virulence of one dengue serotype is enhanced where it co-occurs with another serotype and can successfully invade the same host. This can make predicting the epidemiological outcome of local serotype diversity challenging (Feng and Velasco-Hernández, 1997).

Dengue virus is spread between humans through infected female mosquito bites. The principal vector of dengue, the invasive species *A. aegypti*, is distributed throughout tropical regions (Capinha et al., 2014). This vector is successful in colonising high density human populations, and often adult mosquitoes do not disperse very far, with evidence that some inhabit single households throughout their lives (Stoddard et al., 2013). Although there is some evidence that insecticide treated bed-nets (Lenhart et al., 2008) and curtains (Quintero et al., 2015) may have some protective qualities, as a diurnal vector, the female mosquitoes primarily bite humans and transmit the virus during daylight hours (Canyon et al., 1999). This presents a challenge to dengue management as hosts will be bitten during the day as they move around a landscape thus facilitating rapid movement of the virus.

*A. aegypti* saw a large resurgence at the end of the 20th century and populations continue to grow and increase the global burden of dengue fever following ineffectual control programmes (Gubler, 2002) and rapid urbanisation. The expansion of human settlements simultaneously generates still water breeding sites for *A. aegypti*, provides housing which shelters adult insects and clusters blood meal food sources into a densely populated locale (Gubler, 2011). These conditions are ideal for this urban mosquito to thrive and facilitate dengue outbreaks. This reoccurrence may also have a climate change dimension. As annual temperatures increase in temperate ecosystems, it is likely that *A. aegypti* will follow a shifting climate envelope and further increase the number of humans at risk of this disease (Hales et al., 2002).

A second mosquito, *Aedes albopictus*, is becoming an ever more prevalent vector and is currently the most invasive mosquito (Benedict et al., 2007), capable of spreading multiple diseases as it expands in its global distribution (Medlock et al., 2012). More alarmingly, this vector is also resilient to cooler climates which is facilitating rapid colonisation of temperate regions and subsequent disease transmission of diseases typically confined to the tropics (Rezza, 2012). For example, the 2007 outbreak of chikungunya in Italy, where a strain originating in India was able to spread through the region by invasive *A. albopictus* mosquito bites (Rezza et al., 2007).

Historically, vector control has been the primary approach used to suppress dengue outbreaks. Simply put, reducing mosquito densities can reduce the transmission of the disease as insect densities fall below an entomological threshold limiting the spread of the disease. With increasing interest in control measures, vaccine research is also under way to combat dengue. For example, a chimeric, live-attenuated yellow fever-dengue composite vaccine is currently in phase II b trials (Thavara et al., 2014). However currently there is no vaccine that effectively immunises against every dengue serotype. In fact, the WHO (2015) advocates a combined dengue control approach to make optimal use of available resources within Integrated Vector Management programmes. To this end, our broad aim is to explore vector control and vaccination strategies both in isolation and in combination, for the control of dengue.

Sterile insect technique (SIT) is a biological form of vector control in which sterile insects are released into the environment, compete with wild type insects for mates, and lower the population size through failed reproductive events. We explore a variant on this classic approach to SIT and investigate the use of self-limiting genetic constructs (Alphey et al., 2011). Disease vectors, such as *A. aegypti*, are genetically modified to carry a late-acting, dominantly expressed, lethal mutation (Phuc et al., 2007). Offspring of the resulting crosses do hatch from their egg stage, but later die after the larval density-dependent mortality has removed some individuals from the population. Thus the effect of this form of SIT vector control is not masked by natural density-dependent mortality and significant reductions in adult mosquito population sizes are expected to occur. This in turn means that there are fewer biting vectors and so ultimately, fewer humans should contract dengue. Development of this approach could yield a useful tool to complement established integrated vector management strategies employed to tackle dengue outbreaks.

Given that there are multiple serotypes of dengue virus in circulation, it is essential that detailed attention is given to how hosts are spatially distributed amongst these virus and vector populations. For example, in developing nations the rise in urbanisation and the growth of city population densities is set to favour dengue transmission (Bhatt et al., 2013) and intensive single control programmes may then be less viable in these urban environments. So rather than inhabiting one large homogeneous space, host populations are grouped into distinct patches to represent living in cities and towns that are connected by commuter behaviour. This adds additional realism to understanding dengue epidemiology (Kyle and Harris, 2008) and is particularly important as dengue has been observed to spread primarily through host movements (Tizzoni et al., 2014) rather than mosquito dispersal (Reiner et al., 2014).

The inclusion of vector dynamics results in a system with feedbacks from vector–host and host–vector that make for complex transmission dynamics. We begin by describing, in some detail, the mathematical model before deriving expressions for R0 in a simple landscape. We investigate how disease and/or vector control methods may be synergistically employed across networks of host populations, with a coupled mosquito population and epidemiological model playing out over this landscape. Additionally, we explore the case of imperfectly efficacious controls, with the intention of providing more realistic predictions for seasonal outbreak suppression and whether imperfections in one technology may be tolerated through using a combination of approaches.

## 2 Mathematical model

### 2.1 Epidemiology

We construct a vector–host model as a set of differential equations, where hosts and mosquito dynamics are characterised by the flow of individuals through compartments over time. Here, we give an overview of the model details.

Dengue is a vector-borne disease with viral transmission place between human hosts and infected vector mosquitoes, *X*, during biting events, occurring with rate *a* per mosquito per day, at which point *b*_1_ represents the conversion rate of susceptible individuals, *S*, into infected hosts, *I*. Hence the rate of change of infections follows:

$$\frac{\mathrm{dI}}{\mathrm{dt}}=\frac{{\mathrm{Sab}}_{1}X}{N}.$$

Mass action transmission is assumed in traditional SIR models of infection, where some proportion of encounters between infected and susceptible individuals leads to infection. However this does not adequately capture what is occurring when vectors mediate the transmission of the virus between hosts (McCallum et al., 2001). Transmission through bites does not scale linearly with respect to host population size, instead it saturates for large host populations (Thrall et al., 1995). Hence transmission is independent of host density for a range of host population sizes. This frequency-dependent transmission is best described by scaling the bite rate (*a*) by the host population size (*N*).

We explore the dynamics of dengue when two serotypes are in circulation. This is biologically significant as immune responses against the first serotype to which an individual is exposed generate antibodies that give a temporary cross-immunity to all serotypes. However once this period is over hosts become more susceptible to a secondary infection by a different serotype. These secondary infections are clinically more serious and this is captured in our model through increased mortality, $\alpha_{2}>\alpha_{1}$ and, increased conversion rate, $b_{2}>b_{1}$. These infections often manifest as dengue shock syndrome or dengue haemorrhagic fever.

**Fig. 1:**
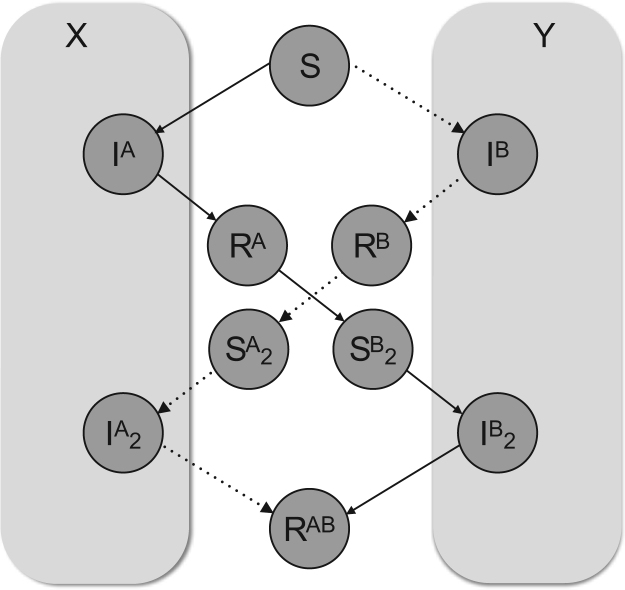
Schematic diagram representing infections through host class infected by two different dengue serotypes. A susceptible host may progress through one of two possible routes, depending on which serotype is carried by the infected vector that first bites them. X mosquitoes carry serotype A, and Y carry B. If bitten by X, hosts will move from the susceptible class, *S*, to primarily infected, *I*^*A*^, and then on to recovered, *R*^*A*^. Then, following a period of cross-immunity , hosts become susceptible to a second serotype, *S*^*B*^_2_, at which point another biting event can move hosts into a secondarily infected class, *I*^*B*^_2_, before another immune response brings them into the fully recovered class, *R*^*AB*^. Circles within the grey rectangles indicate that progression to these classes is dependent on interactions with vectors.

To describe the dynamics of these secondary infections, additional classes were added to the model. Rather than simply one infection type occurring, any given susceptible host may be bitten by an infected mosquito, *X*, carrying serotype A or another mosquito, *Y*, carrying serotype B. Thus from an individual host's perspective, if they are bitten at least once they will track through one of two trajectories, as illustrated in Fig. 1.

Mirroring the transmission from vector–host, the host–vector transmission relies on the per mosquito bite rate, *a*, the subsequent conversion of adult mosquitoes into vectors as they become infectious, *c*, the number of mature mosquitoes, *M*, and infected host density, I/N, hence the dynamics of infected vectors (*X*) follows:

$$\frac{\mathrm{dX}}{\mathrm{dt}}=\frac{\mathrm{acMI}}{N}.$$

Importantly, experimentally it has been shown that infected *Aedes* mosquito cells resist viral entry of another serotype (Dittmar et al., 1982), hence we assume here that mosquitoes can carry either serotype, but not both.

### 2.2 Control methods

Successful control interventions must reduce the severity of a dengue epidemic by lowering the number of infected hosts and reducing virus transmission. In this model, two control strategies are considered, which reduce the size of the host susceptible and mosquito juvenile classes. The first method described, vaccination, directly targets the host infection dynamics. Control of host infections may also be achieved by targeting the vector population through SIT methods.

Susceptible hosts can be targeted by a vaccination program which moves these individuals straight into a recovered class. This bypasses the infection stages to reduce the overall burden of the disease over the course of the season. Although under development, currently there is no availability of sufficiently effective multivalent dengue vaccines. The hypothetical vaccine employed here immunises against both strains, with imperfections also investigated where the vaccine is only efficacious against one of the serotypes. A straightforward binary operator is used to capture which host class would be (proportionately) vaccinated; for a perfect vaccine, which provides full protection to both serotypes, vaccinated individuals were added to the fully recovered class *R* at a rate of: ζγS. Alternatively, vaccines could provide immunity to serotype B but only temporary cross-immunity to serotype A. Once cross-immunity expires these individuals can be infected with serotype A, so they cannot be placed in the fully recovered class. Instead the temporary recovered class, *R*^*B*^, is added to at a rate of (1−ζ)γS. Hence setting *ζ* to 0 or 1 changes the outcome of vaccination controls.

Vector control provides an alternative perspective from which we approach the challenge and public health control of dengue. Mosquito dynamics were modelled with individuals maturing from a juvenile class, *J*, into mature biting adults, *M*, which could subsequently become a vector of serotypes *A* or *B* with mosquito classes *X* or *Y*, respectively. In our model juvenile mosquitoes (*J*) experience density-dependent mortality through a one-parameter density-dependent function (Bellows, 1981). This is a simple way of capturing vector ecology where *δ* moderates the strength of this density-dependent regulation:

f(J)=−Jln(1+δJ).

We separate out density-dependent mortality and genetic-based SIT control as these are mechanistically distinct. Late-acting lethal mutations only occur after density-dependent ecological processes have played out, resulting in additional reductions to the juvenile population. Such vector control technology could be achieved through genetic modification techniques to generate self-limiting genetic constructs (Phuc et al., 2007). This sort of vector control enters the model through effects on vector births. If the breeding mosquito population is Z=M+X+Y, then under SIT, increases in the juvenile population follow:

$$h(J)=\mathrm{gZ}\frac{Z}{Z+\Psi }.$$

Here *g* represents the intrinsic rate of increase of the insect population in the absence of intraspecific competition. SIT control is introduced through Ψ, which denotes the number of genetically modified insects released at the start of the season. Where no modified insects are released this equation simply denotes a linear juvenile birth rate with respect to the number of adults. As more sterile insects are released successful wildtype mating attempts become rarer resulting in a lower rate of juvenile production.

In reality the genetically engineered lethality gene may be unstable and not always cause mortality in vectors expressing it. In this case some offspring who inherit the lethal gene do not die. To model this, the juvenile birth rate was expanded to:

$$k(J)=\mathrm{gZ}(\frac{Z}{Z+\Psi }+(1-\epsilon )\frac{\Psi }{Z+\Psi }).$$

Here, if ϵ=1 then the equation collapses to the perfect control situation. To alter the effectiveness of genetic control, the degree of lethality can take a range of values between 0 and 1. The full equation governing the juvenile population (described in Section 2.4) combines density-dependent mortality, the SIT-moderated births described here and a developmental rate as insects mature into adults.

### 2.3 Creating a network structure

Here we explore small networks of up to five patches, where one central hub connects all other satellite patches to it in a hub-spoke configuration. Differences in patch sizes can represent size differences among cities and towns, as illustrated in the supplementary information. Network structures were influenced by changing the commuter flow between these patches, whilst mosquitoes were assumed to be only locally distributed and hence could not migrate between patches.

So far the model equations have described details pertaining to a single homogeneous environment. We expand the mathematical framework to a patch network of human and mosquito populations connected by commuting hosts. A susceptible host individual has two mutually exclusive potential routes of infection, either by remaining in their patch, *i*, during the day and being bitten by these mosquitoes, or by commuting to patch *j* and being exposed to bites in this other patch. Hence despite no net movement of individuals over time, infections can be picked up by commuting individuals and spread back to their original patch.

Whilst individual patches have their own infection dynamics and local mosquito population dynamics, a connectivity matrix generates a network of interactions between these patches. The *ω*_*ij*_ commuter flow term describes directional commutes from patch *j* to *i*, referring to one-way daily commutes from a town to the city patch. A second commuter behaviour, bidirectionality, refers to commuting occurring both from towns to the city and from the city to the towns. This reciprocal commuting direction is included with the addition of the *ω*_*ji*_ term.

More movement terms are introduced to determine the proportion of each host class that commutes. Hence, *m*_*S*_, *m*_*I*_, *m*_*R*_ and $m_{I_{2}}$ represent the proportion of classes S, I, R and *I*_2_, respectively, that undergo a daily commute. These proportions were fixed to the same value for most of the analysis, the exception being secondarily infected individuals, who owing to the severity of their condition, were assumed to commute far less (in fact, an order of magnitude less than the other classes).

Given that during the day the actual population sizes of each patch vary according to commuter dynamics, the population size alone does not inform the frequency dependence in transmission. Instead, *Ω*_*i*_ is incorporated into the daily population size, which is simply a summation of the flow into patch *i* from other patches, to determine the net change to daily population sizes.

Hence, between connectivity and class movement terms, the network could be set up with infections of non-commuting hosts being gained in patch *i* following:

$$f(I)=(1-\omega_{\mathrm{ji}}m_{S})\frac{S_{i}a_{i}b_{1}X_{i}}{N_{i}+\Omega_{i}}.$$

While commuting hosts become infected following:

$$g(I)=(\omega_{\mathrm{ji}}m_{S})\frac{S_{i}a_{j}b_{1}X_{j}}{N_{j}+\Omega_{j}}.$$

This is then expanded to generate a five patch network, arranged in a wheel-spoke design. This means that the central city patch, *i*, connects to every other satellite (town) patch in the network. In analysing this model, epidemics are assumed to originate in the city, which is in keeping with epidemiological information on dengue infections (Cummings et al., 2004).

### 2.4 Full model equations

Based on all this biology, we now present the full model for dengue transmission over a network of *n* patches from the perspective of the *i*th patch, capturing two serotypes of dengue virus, frequency-dependent transmission between vectors and hosts, density-dependent mosquito population growth, a period of cross-immunity following infection and antibody-dependent enhancement in secondary cases of infection. Overlaid are control terms, both through vaccination and vector control, which can be varied by patches i,j,k,l and *m* across the five patch network. Eqs. (1), (2), (3), (4), (5), (6), (7), (8), (9), (10) refer to human population dynamic variables, (11), (12), (13), (14) to mosquito population dynamics, and (15), (16), (17) capture total population sizes. All state variables and parameters are fully described in Table 1, Table 2.

#### 2.4.1 Human host dynamics

In this section we describe, in full, the epidemiological model for infection dynamics in (human) hosts. Eqs. (1), (2), (3) represent a susceptible-infected-recovered (SIR) process with frequency dependent transmission of dengue as infected mosquitoes bite susceptible hosts, converting them into infected hosts, who then recover at a rate of *ρ*_1_:

(1) dSidt=−(1−∑j=1j≠inωjimS)Siab1(Xi+Yi)Ni+Ωi−∑j=1j≠inωjimSSiab1(Xj+Yj)Nj+Ωj−γiSi

(2) dIiAdt=(1−∑j=1j≠inωjimS)Siab1XiNi+Ωi+∑j=1j≠inωjimSSiab1XjNj+Ωj−IiA(α1+ρ1)

(3) $$\frac{{\mathrm{dR}}_{i}^{A}}{\mathrm{dt}}=\rho_{1}I_{i}^{A}-\eta R_{i}^{A}$$

The terms ∑j=1j≠inωjimS represent a summation of all hosts that commute to every other patch and are therefore subject to biting events from mosquitoes in patches other than the focal patch *i*. This means that in Eq. (2), patch *i* infections are acquired both by infected vectors in patch *i* biting susceptible hosts who do not commute, (1−∑j=1j≠inωjimS)Siab1XiNi+Ωi, and also as a result of biting events occurring in other patches on commuting hosts, ∑j=1j≠inωjimSSiab1XjNj+Ωj. Daily commutes modify patch sizes, hence *N*_*j*_ is modified by the addition of *Ω*_*j*_, which updates population sizes to allow the model to calculate frequency-dependent transmission events. Susceptible hosts are also removed from Eq. (1) through vaccination, determined by $\gamma_{i}S_{i}$:

(4) dSiB2dt=ηRiA−(1−∑j=1j≠inωjimR)SiB2ab2YiNi+Ωi−∑j=1j≠inωjimRSiB2ab2YjNj+Ωj

(5) dIiB2dt=(1−∑j=1j≠inωjimR)SiB2ab2YiNi+Ωi+∑j=1j≠inωjimRSiB2ab2YjNj+Ωj−IiB2(α2+ρ2)

Eq. (4) models the transition of hosts who have recovered from one serotype of dengue, A, back into a secondarily susceptible class after a period of cross-immunity, captured by $\eta R_{i}^{A}$. Hence in Eq. (5) secondary infections are acquired where these hosts are bitten by mosquitoes carrying a second serotype, B, with subsequent loss from this class, $I_{i}^{B_{2}}(\alpha_{2}+\rho_{2})$, arising through mortality and recovery, respectively.

Alternatively, host may progress through a second route of infection depending on which serotype they are first infected with. If B is acquired first, then Eqs. (6), (7), (8), (9) will occur with Eqs. (2), (3), (4), (5):

(6) dIiBdt=(1−∑j=1j≠inωjimS)Siab1YiNi+Ωi+∑j=1j≠inωjimSSiab1YjNj+Ωj−IiB(α1+ρ1)

(7) $$\frac{{\mathrm{dR}}_{i}^{B}}{\mathrm{dt}}=\rho_{1}I_{i}^{B}-\eta R_{i}^{B}+(1-\zeta )\gamma_{i}S_{i}$$

(8) dSiA2dt=ηRiB−(1−∑j=1j≠inωjimR)SiA2ab2XiNi+Ωi−∑j=1j≠inωjimRSiA2ab2XjNj+Ωj

(9) dIiA2dt=(1−∑j=1j≠inωjimR)SiA2ab2XiNi+Ωi+∑j=1j≠inωjimRSiA2ab2XjNj+Ωj−IiA2(α2+ρ2)

(10) $$\frac{{\mathrm{dR}}_{i}}{\mathrm{dt}}=\rho_{2}(I_{i}^{B_{2}}+I_{i}^{A_{2}})+\zeta \gamma_{i}S_{i}$$

One important difference arises in Eq. (7) (cf. Eq. (3)) with the inclusion of an additional term: $(1-\zeta )\gamma_{i}S_{i}$. Vaccines against only one serotype will push susceptible hosts into this class and may indirectly contribute to the $S_{i}^{A_{2}}$ pool. However, vaccines against both serotypes transfer hosts in Eq. (1) straight to Eq. (10) thereby by-passing infectious classes entirely. Hence in this class, numbers of fully recovered hosts (Eq. (10)) change with vaccination, $\zeta \gamma_{i}S_{i}$, and as secondarily infected hosts recover, $\rho_{2}(I_{i}^{B_{2}}+I_{i}^{A_{2}})$.

#### 2.4.2 Mosquito population dynamics

In this section we present the details of the mosquito population dynamics. We consider a structured life cycle (juveniles and adults) through which mosquitoes develop. Juvenile mosquito numbers increase through births $\left({\mathrm{gZ}}_{i}(\frac{Z_{i}}{Z_{i}+\Psi_{i}}+(1-\epsilon )\frac{\Psi_{i}}{Z_{i}+\Psi_{i}}\right)$, where ${\mathrm{gZ}}_{i}$ laid eggs fail to develop as a result of modified mosquito release $\frac{Z_{i}}{Z_{i}+\Psi_{i}}$, accounting for gene lethality of less than 100% with $(1-\epsilon )\frac{\Psi_{i}}{Z_{i}+\Psi_{i}})$. Juveniles numbers decrease through development into mature adults (Eqs. (11), (12)) at rate *ϕ* and die through the processes of density dependent intraspecific competition $\left(\ln(1+\delta J_{i})\right)$. Adult mosquitoes numbers increase through maturation of immature mosquitoes and decrease through a density-independent deaths (at rate $\mu M_{i}$). The conversion of mosquitoes into infectious vectors is characterised by Eqs. (13), (14):

(11) $$\frac{{\mathrm{dJ}}_{i}}{\mathrm{dt}}={\mathrm{gZ}}_{i}(\frac{Z_{i}}{Z_{i}+\Psi_{i}}+(1-\epsilon )\frac{\Psi_{i}}{Z_{i}+\Psi_{i}})-J_{i}\ln(1+\delta J_{i})-\phi J_{i}$$

(12) dMidt=ϕJi−μMi−acMiNi+Ωi((IiA+IiB)(1−∑j=1j≠inωjimI)+(IiA2+IiB2)(1−∑j=1j≠inωjimI2))−acMiNj+Ωj(∑j=1j≠inωij(mI(IjA+IjB)+mI2(IjA2+IjB2)))

Dengue transmission from humans to mosquitoes is detailed in Eqs. (13), (14). Within patch *i*, mosquitoes acquire infections, $\frac{{\mathrm{acM}}_{i}}{N_{i}+\Omega_{i}}$, by biting primary and secondarily infected hosts that stay in the patch, (IiA(1−∑j=1j≠inωjimI)+IiA2(1−∑j=1j≠inωjimI2)), or that have commuted into the patch, ∑j=1j≠inωij(mIIjA+mI2IjA2).

(13) dXidt=acMiNi+Ωi(IiA(1−∑j=1j≠inωjimI)+IiA2(1−∑j=1j≠inωjimI2))+acMiNi+Ωi∑j=1j≠inωij(mIIjA+mI2IjA2)−μXi

(14) dYidt=acMiNi+Ωi(IiB(1−∑j=1j≠inωjimI)+IiB2(1−∑j=1j≠inωjimI2))+acMiNi+Ωi∑j=1j≠inωij(mIIjB+mI2IjB2)−μYi

The human population size per patch was modelled as:

(15) $$N_{i}=S_{i}+I_{i}^{A}+R_{i}^{A}+S_{i}^{B_{2}}+I_{i}^{B_{2}}+I_{i}^{B}+R_{i}^{B}+S_{i}^{A_{2}}+I_{i}^{A_{2}}+R_{i}$$

And the breeding vector population by:

(16) $$Z_{i}=M_{i}+X_{i}+Y_{i}$$

#### 2.4.3 Human host movement

Finally, the net human flux caused by daily commuting into patch *i* from surrounding patches is modelled as:

(17) Ωi=∑j=1j≠inωij(mSSj+mI(IjA+IjB)+mR(RjA+RjB+SjA2+SjB2+Rj)+mI2(IjA2+IjB2))−ωji(mSSi+mI(IiA+IiB)+mR(RiA+RiB+SiA2+SiB2+Ri)+mI2(IiA2+IiB2))

This equation accounts for human movement changing the size of patches during the day, which impacts on frequency dependent transmission events. This flux is contingent on heterogeneous patch connectivity (as the central patch has more connections than the others) and on the differential movement of healthy and infected individuals, thus is a dynamic measure that varies as infection spreads through the network.

### 2.5 Numerical methods

Simulations were undertaken using a 4th order Runge–Kutta function (implemented in R version 3.2.2) integrating 70 dynamic variables over 150 iterations, reflecting the seasonal period investigated of 150 days. Over each model iteration infection began with one infected human for each of the two serotypes, located in the central city patch. Infected mosquito numbers were initially set to 0.1% of the adult vector population. Then the numbers of primary and secondary host infections were analysed across the entire network over the course of a season.

The geometric mean number of infected hosts carrying serotype A was used to compare the results of simulations under different starting conditions. Whilst summations were also explored, geometric means were deemed the most appropriate metric to use. This is because they allow averages to be taken over periods of time and are useful for comparing sets of numbers with different ranges of values, acknowledging that cities would produce far more infections than towns, owing to differences in population sizes.

## 3 Basic reproductive number (R0) analysis

The basic reproductive number (R0) defines the number of infections that arise as a result of single infected individual (e.g. Diekmann, 1990; Diekmann et al., 2010). For a vector-borne disease this involves determining expressions based on disease spread from vector-to-host, $R0_{V\to H}$, and host-to-vector transmission rates, $R0_{H\to V}$, with the overall R0 for the vector-borne disease given by the product of $R0_{V\to H}\cdot R0_{H\to V}$.

The component R0 expressions (vector-to-host, $R0_{V\to H}$, and host-to-vector $R0_{H\to V}$) for dengue disease spread in a two patch environment are given by (see supplementary information for the full derivation):

(18) $$R0_{V\to H}=\frac{1}{2\mu }(\overset{˙}{I_{\mathrm{ii}}}+\overset{˙}{I_{\mathrm{jj}}}+|\sqrt{{\left(\overset{˙}{I_{\mathrm{ii}}}-\overset{˙}{I_{\mathrm{jj}}}\right)}^{2}+4\overset{˙}{I_{\mathrm{ij}}}\overset{˙}{I_{\mathrm{ji}}}}|)$$

(19) $$R0_{H\to V}=\frac{1}{2(\alpha_{1}+\rho_{1})}(\overset{˙}{X_{\mathrm{ii}}}+\overset{˙}{X_{\mathrm{jj}}}+|\sqrt{{\left(\overset{˙}{X_{\mathrm{ii}}}-\overset{˙}{X_{\mathrm{jj}}}\right)}^{2}+4\overset{˙}{X_{\mathrm{ij}}}\overset{˙}{X_{\mathrm{ji}}}}|)$$

where infection rates, say $\overset{˙}{I_{\gamma \delta }}$, correspond to transmission events occurring in patch *δ* that contribute to infections in *γ*. These rates governing infection dynamics can be substituted into Eqs. (18), (19) to generate the full (component) R0 expressions:

(20) $$R0_{V\to H}=\frac{1}{2\mu }(\frac{{\mathrm{ab}}_{1}(1-\omega_{\mathrm{ji}}m_{S})N_{i}}{N_{i}+\Omega_{i}}X_{i}+\frac{{\mathrm{ab}}_{1}(1-\omega_{\mathrm{ij}}m_{S})N_{j}}{N_{j}-\Omega_{i}}X_{j}\;+|\sqrt{{\left(\frac{{\mathrm{ab}}_{1}(1-\omega_{\mathrm{ji}}m_{S})N_{i}}{N_{i}+\Omega_{i}}X_{i}-\frac{{\mathrm{ab}}_{1}(1-\omega_{\mathrm{ij}}m_{S})N_{j}}{N_{j}-\Omega_{i}}X_{j}\right)}^{2}+\frac{4a^{2}b_{1}^{2}m_{S}^{2}\omega_{\mathrm{ji}}N_{i}X_{j}\omega_{\mathrm{ij}}N_{j}X_{i}}{\left(N_{j}-\Omega_{i})(N_{i}+\Omega_{i}\right)}}|).$$

(21) $$R0_{H\to V}=\frac{1}{2(\alpha_{1}+\rho_{1})}(\frac{{\mathrm{acM}}_{i}(1-\omega_{\mathrm{ji}}m_{I})}{N_{i}+\Omega_{i}}I_{i}+\frac{{\mathrm{acM}}_{j}(1-\omega_{\mathrm{ij}}m_{I})}{N_{j}-\Omega_{i}}I_{j}\;+|\sqrt{{\left(\frac{{\mathrm{acM}}_{i}(1-\omega_{\mathrm{ji}}m_{I})}{N_{i}+\Omega_{i}}I_{i}-\frac{{\mathrm{acM}}_{j}(1-\omega_{\mathrm{ij}}m_{I})}{N_{j}-\Omega_{i}}I_{j}\right)}^{2}+\frac{4a^{2}c^{2}m_{I}^{2}M_{i}\omega_{\mathrm{ij}}I_{j}M_{j}\omega_{\mathrm{ji}}I_{i}}{\left(N_{j}-\Omega_{i})(N_{i}+\Omega_{i}\right)}}|).$$

For vector-borne disease dynamics, a set of different starting conditions can be applied to the general R0 model to compare the number of secondary cases generated per primary case for different epidemiological scenarios. In the following analysis, infections of both vectors and hosts are initiated in patch *i*, but the connectivity between patches *i* and *j* may vary. Hence *I*_*i*_=1, *I*_*j*_=0, *X*_*i*_=1 and *X*_*j*_=0. We make the further simplifying assumption that infection has a negligible effect on the movement of hosts at the beginning of the epidemic, hence *m*_*S*_=*m*_*I*_. Therefore in a two patch system, the total change in population size in patch i during the day, *Ω*_*i*_, is governed by the equation:

(22) $$\Omega_{i}=\omega_{\mathrm{ij}}m_{S}N_{j}-\omega_{\mathrm{ji}}m_{S}N_{i}.$$

Now, by substituting connectivity parameters corresponding to different commuter behaviours into Eqs. (20), (21), (22) and using the overall expression that $R0=R0_{V\to H}.R0_{H\to V}$, a set of simplified R0 expressions can be derived (Table 3).

Under different commuter behaviours (Table 3), the R0 expression can be further shown to depend on vector biology and conditions associated with the host population size:

(23) $$R0=\frac{a^{2}b_{1}{\mathrm{cM}}_{i}}{\mu (\alpha_{1}+\rho_{1})}\cdot f(\mathrm{host}\;\mathrm{population}\;\mathrm{size}\;\mathrm{terms}).$$

where rates of conversion of bitten susceptible hosts into the infected class, *b*_1_, conversion of mature mosquitoes into infectious vectors, *c*, the number of mature mosquitoes, *M*_*i*_ and biting rate, *a*, increase the value of R0. Whereas rates of vector mortality, *μ*, infected host mortality, *α*_1_ and recovery, *ρ*_1_, are parameters that reduce the spread of disease.

The function associated with host population size is altered by connectivity terms *ω*_*ij*_ and *ω*_*ji*_. As more complex commuter behaviours are permitted, R0 becomes increasingly more complex to accommodate the daily fluctuations in population size that occur across the network. The simplest R0 presented, for isolated, non-commuting patches, demonstrates why this is important. Within patch *i*, the ratio of vectors to hosts, $\frac{M_{i}}{N_{i}}$, impacts on the spread of disease. As the number of hosts increases, the density of vectors per host decreases and fewer bite events can occur. This result is due to the frequency dependent transmission of vector borne diseases. As commuter behaviour changes this alter daily population sizes, and R0 then incorporates associated terms such as the size of patch *j*, *N*_*j*_, and the movement coefficient *m*_*S*_.

**Fig. 2:**
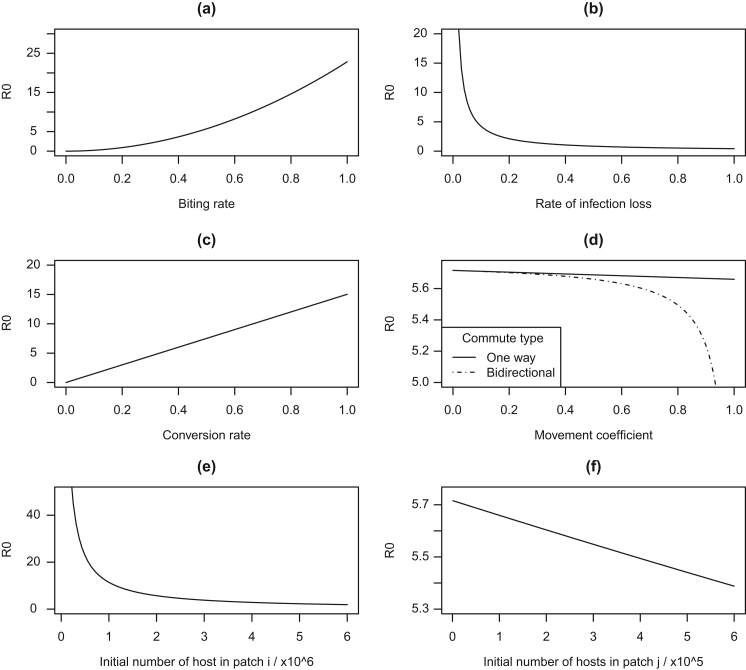
Effects of vector biology and host behaviour on R0. (a) Vector biting rate, *a*. (b) The rate of vector mortality *μ* (the curve is identical for host mortality *α*_1_ and recovery, *ρ*_1_). (c) Conversion rate of susceptible to infected hosts, *b*_1_ (vector conversion rate, *c* and the number of vectors in patch *i*, *M*_*i*_ produce the same linear relationship). (d) The movement coefficient parameter, *m*_*S*_, behaves differently between one-way and bidirectional movement. (e) and (f) explore the impact of different patch population sizes in *i* and *j*, *N*_*i*_ and *N*_*j*_, respectively.

Fig. 2 illustrates the changes in R0 with respect to a range of vector biology and host behaviour. Unless stated otherwise, the one way commute system is used (as for most parameters there was no difference among the three R0 expressions displayed in Table 3). Using these we make predictions about how the epidemic will initially spread from one infected host in response to different ecological and epidemiological parameters. Increasing the biting rate, *a*, will lead to a non-linear increase in R0, whereas increasing host conversion, *b*_1_, increases R0 in a linear way. The effect of the movement coefficient, *m*_*S*_, varies between different R0 models, becoming more influential for bidirectional behaviour as it impacts the commuting populations of both patches and can lead to proportionate decreases in R0.

## 4 Network disease dynamics

**Fig. 3:**
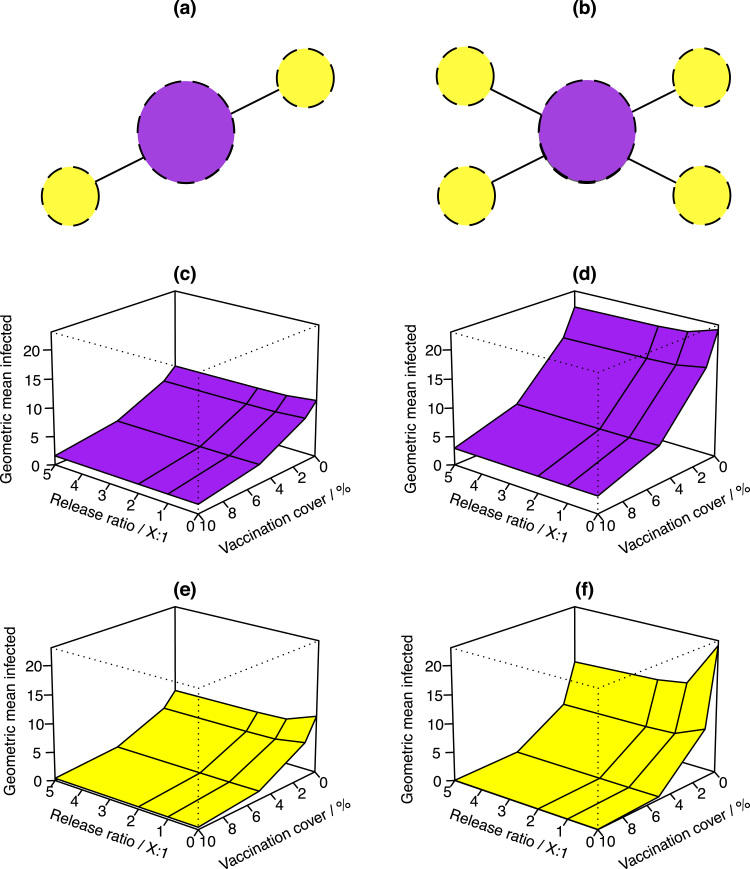
3-D surfaces illustrating the effect of spatially explicit controls. Two satellite (a) and four satellite (b) network configurations are displayed, corresponding to results (c) and (e), and (d) and (f), respectively. Controls are applied only in the city patch (c) and (d) or satellite town patches (e) and (f). Through both increasing the percentage of the population that is vaccinated, and releasing more sterile insects relative to the wild vector population (ratio X:1, sterile:wild-type), greater reductions to the numbers of infections are observed.

The effectiveness of different spatially explicit control applications was examined through varying which patches amongst the network received control. Fig. 3 displays the network configurations explored, in which a large central patch is connected to either two (Fig. 3a) or four (Fig. 3b) satellite patches. As shown in Fig. 3, both insect releases and vaccination independently show saturating effectiveness for control. Hence, the greatest reductions in cases per unit effort occur in systems that have little to no control already in place. Both control types can also act synergistically. A consequence of these two observations is that any pre-existing control strategy using just one type of control will greatly benefit from adding even a small amount of the other control type.

Town-only control measures apply controls to the satellite patches only. As shown in Fig. 3c and Fig 3d, under this strategy the dengue epidemic is brought completely under control, where the mean numbers of infected hosts reach zero under high levels of vaccination. This did not occur for city-only controls, applied only to the central network patch, operating over this same range of values. So town-only control may not only be more effective, but also potentially more efficient than city-only controls. In our simulations, this is because for a vaccination programme covering 10% of the population, city-only control would require vaccinating 200,000 people, whereas for town-only measures the maximum number needing vaccination over the largest network explored is only 4000. The actual number of insects that should be released is similarly reduced by several orders of magnitude when switching from city-only to town-only control.

**Fig. 4:**
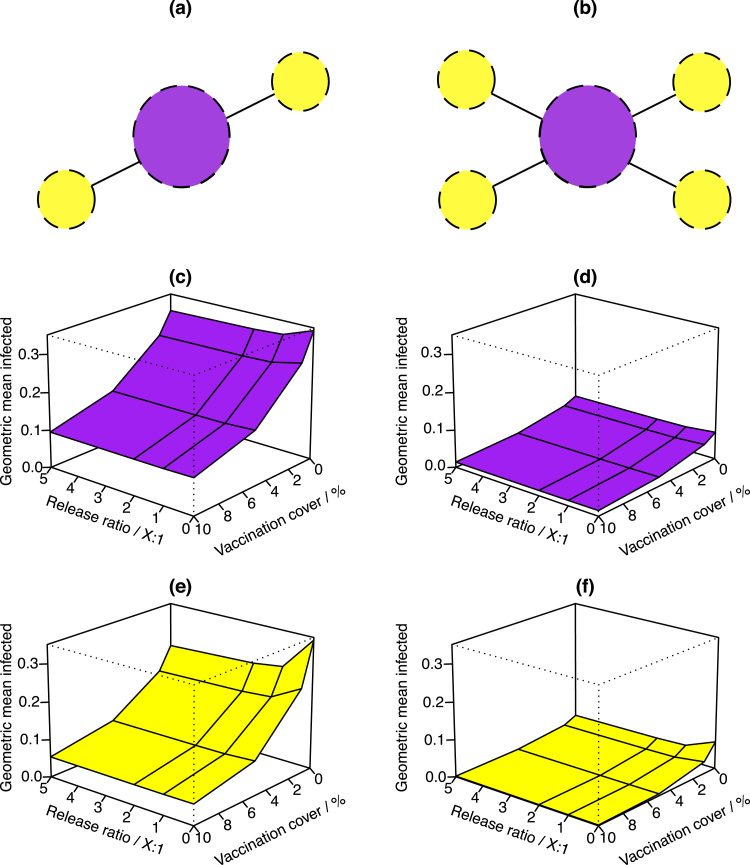
3-D surfaces illustrating the effect of spatially explicit control application on secondary dengue. Two satellite (a) and four satellite (b) network configurations are displayed, corresponding to results (c) and (e), and (d) and (f), respectively. Controls are applied only in the city patches (c) and (d) or satellite town patches (e) and (f). Both increased vaccine coverage and increased sterile insect release lead to reductions in secondary cases.

When comparing the smaller network to the larger one, for example Fig. 3a and Fig. 3b, it is clear that the number of cases increases across the larger network when all other variables are kept the same. So the more patches connected to a large central patch, the larger the overall susceptible pool and dengue can spread to more hosts. However, as Fig. 4 demonstrates, the converse is true for secondary cases, which are reduced in the large network relative to the small network.

This difference in primary and secondary dengue responses to increasing network size is predicted to be due to a herd immunity response. On the larger network there is a greater increase in city population size during the day, due to increased commuting from a larger number of towns. In this model secondary dengue is the result of a host being bitten by two different mosquitoes, each bearing a different serotype, provided enough time elapses between each event for cross-immunity to be lost. On a larger network the probability of any one individual in the city being bitten twice is reduced, as a herd immunity effect provides some relative protection. This phenomenon is common to each spatial control application, indicating that this is not an artefact of control strategies and instead, a result of network configuration.

However, both primary and secondary cases of dengue respond similarly under different spatial controls. It seems that by reducing the spread of infection to towns in a town-only control type, large scale reductions result that exceeds the effectiveness of control focussed in the high population, central city patch. This counter-intuitive result is further explored by altering commuter flow behaviour (see supplementary information).

## 5 Imperfect controls

**Fig. 5:**
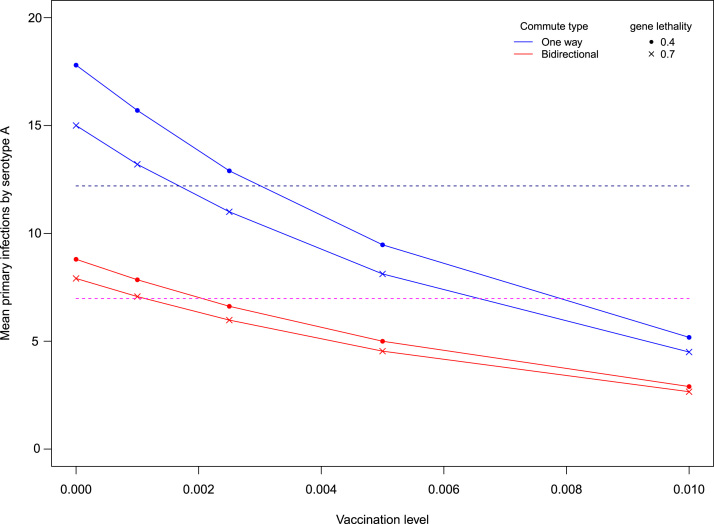
Compensating imperfect vector control. The results for perfectly lethal genetic control provide a baseline for comparison (dotted lines), where modified insects were released at every patch in a 2:1 ratio with the wild-type insects. Supplementing an imperfect vector control with vaccinations can offset the increase in infections that arise from reduced control lethality.

Having established how commute type, network size and control combinations interact to determine the best control methods, in this section we investigate the efficacy of these controls. So far it has been assumed that vaccines are entirely efficacious and that the introduced lethality gene is entirely stable, hence 100% lethal. Fig. 5 shows the results of relaxing the assumption of perfect control.

In Fig. 5 the increase in infections can be seen as the difference between the baseline for perfect controls and the lines at Vaccinationlevel=0. As more hosts are vaccinated, the mean number of infections can be brought down to intersect with the baseline. This indicates that the increase in infection associated with imperfect genetic techniques can be compensated. Here this is achieved by sustaining fairly low levels of vaccination, even for genes with only a 40% lethality rate. Fig. 5 shows primary infections, but the pattern holds true for secondary cases as well.

In contrast, imperfect vaccinations give a different result. Here we assume that imperfect vaccination renders individuals immune to one serotype but not to the other co-circulating serotype. Given that there is no currently used multivalent vaccine, this is a situation that could easily arise if vaccine programmes do not match up with the serotypes that are circulating a particular region. Commuting into regions with different strains circulating to those that an individual has been vaccinated against could also give rise to this situation.

**Fig. 6:**
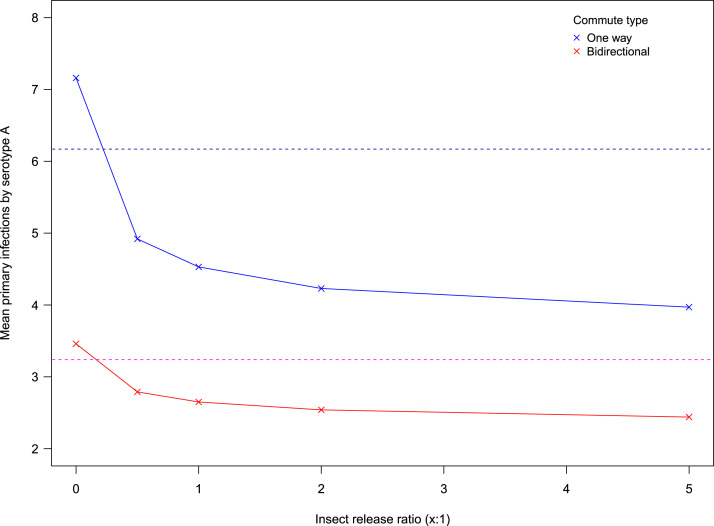
Compensating imperfect vaccines. The dashed lines display the effectiveness of a hypothetical, fully cross-protective vaccine applied to 1% of the population. Where one serotype (A) escapes vaccine control, the resulting increase in primary infections can be offset by insect control.

As Fig. 6 illustrates, low levels of perfect vector control can offset the increased primary infections brought about by imperfectly vaccinating a very small fraction (1%) of each population in the network.

**Fig. 7:**
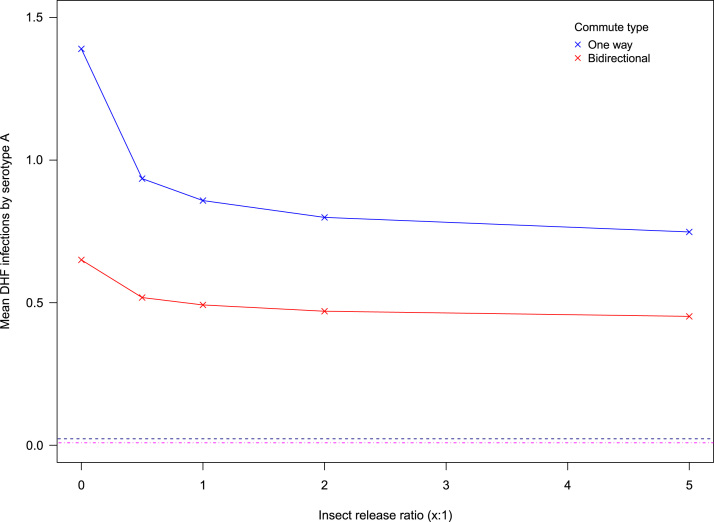
Failure to compensate imperfect vaccines (set at 1% population coverage in every patch) for secondary cases. The low number of infections brought about by a perfect vaccine baseline (dotted lines) cannot be achieved by strategies using an imperfect vaccine in conjunction with vector control.

However, secondary cases see a very large increase that cannot be compensated by vector control, as is illustrated in Fig. 7.

Given that these vaccines indirectly increase the size of the secondarily susceptible class this makes sense. However, this means that attempting to employ vaccines that are not efficacious against any one serotype circulating a region will vastly increase the mean number of secondary cases associated with that serotype, as compared to a perfectly controlled situation.

Sensitivity analysis was carried out on these results, which revealed that vector population dynamic and infectious parameters were crucial in determining the severity of dengue outbreaks. In particular, increases in vector bite rate and longevity lead to proportionately more secondary dengue cases than other model parameters. Details are included in the supplementary material.

## 6 Discussion

Here, we investigate the combined effects of vector control and vaccination on dengue epidemics in small networks. Our results illustrate that the movement of humans around a landscape influences the transmission of dengue. This general idea is compatible with previous work, for example, Evans and Bishop (2014) employ a spatial dengue model with pulsed vector control releases. Consistent with our results, they found that increasing the number of sites and frequency of vector control increases effectiveness, but that programs become less effective for higher insect densities. Furthermore, vaccines and vector control can act synergistically, and the effectiveness of either strategy is dependent on network size (Fig. 3, Fig. 4). This result could be investigated further by adding more patches to the network, allowing heterogeneities in parameter values to be explored. For example, this approach could reflect differences in mosquito larval development rates among patches with different abiotic conditions, as this parameter is associated with local rainfall and temperatures.

Consistent with these theoretical findings, Nevai and Soewono (2014) examine a hub-spoke network with a similar emphasis on the role of movement and spatial heterogeneity. However, their model does not incorporate dengue epidemiology features such as multiple serotypes or host recovery, which as we have shown, yields more detailed insights into the relative effectiveness of controls on primary versus secondary cases. This is particularly relevant where interactions between serotypes increase the incidence of secondary infections, which should be accounted for in any control strategy.

A more nuanced result of our analysis is that under certain conditions control strategies targeting smaller patches rather than the large patch are more effective, hence control may be more cost effective to employ and more likely to be achieved. So depending on commuter flow, vaccination thresholds are more likely to meet their quotas and fewer genetically modified insects may need to be released across the network. Differences in commuter flow are important in determining the control of dengue, with both the numbers moving and the directions of commutes influencing the ratios of vectors to hosts among patches. Simulation results are included in the supplementary information which recapitulate predictions made from the R0 analysis relating to the role of this ratio in reducing the burden of disease (Fig. 2). As a management tool, this model could suggest which patches should be controlled to achieve the greatest reduction in disease cases. To achieve this, further expansion of the analysis undertaken here would be required to incorporate a cost–benefit analysis, which could determine an economic basis for different management strategies produced by this epidemiological model.

More realistic conclusions can be drawn about intervention effectiveness by exploring the assumptions of perfect controls. Current control methods are imperfect by nature, but as shown in Fig. 5, inefficiencies in the genetic modification of vectors can easily be compensated by perfect vaccines. Whilst imperfect vaccines can be compensated by insect releases for primary infections (Fig. 6), this is not possible for severe secondary cases (Fig. 7). Imperfect control measures have also been explored in Thavara et al. (2014) and these findings are mostly consistent with this study on the use of imperfect vaccinations. Results differ here in that our research did not find that vector control could increase disease transmission in areas of high mosquito abundance. However, their individual based model uses a homogeneous environment and does not explicitly model vector dynamics, which may explain these differences in our findings. These differences suggest that the emergent properties of the vector population dynamics that have been modelled here prevent this outcome. Indeed, sensitivity analysis highlights that vector ecology and specific mosquito life history characteristics are vital in determining the severity of dengue epidemics. Following simpler Ross–MacDonald formulations (e.g. Smith et al., 2012), the most important vector parameters are biting rate and vector longevity (supplementary information). To test the predictive power of our model by integrating real data, it would be crucial to have accurate measures for these parameters in the field, as slight changes are expected to have large effects on the dynamics of the disease.

In summary, it seems that small networks capturing human movement allow for an in-depth analysis of the transmission of dengue fever. Such an analysis could be used to inform the most effective spatial application of control strategies which, in accordance with these results, should take commuter dynamics into consideration. In addition, vector biology has a large impact on the outcome of the disease, so insect ecology should be fully incorporated into this human health problem by considering mosquito population dynamics. Finally, focus on imperfectly efficacious controls indicates that in reality some strategies may be less effective than would be expected; such methods can even increase the incidence of severe dengue due to serotype interactions. Thus for maximum impact only controls that are appropriate for regional virus diversity should be applied to population networks, ensuring that any employed vaccine is effective against every circulating serotype. This may be particularly relevant in the context of poorer countries where dengue is a growing issue and so, efficient, cost-effective control is a major priority.

## Competing interests

None declared.

## Figures and Tables

**Table 1:** State variable table.

Symbol	Description	Initial value in city	Initial value in town
$N_{i}$	Total host population size	2,000,002	10,000
*S*_*i*_	Susceptible hosts	2,000,000	10,000
$I_{i}^{A},I_{i}^{B}$	Infected hosts (with serotype A, B)	1, 1	0
$R_{i}^{A},R_{i}^{B}$	Hosts that have recovered from a primary infection	0	0
$S_{i}^{A},S_{i}^{B}$	Recovered hosts that have lost cross-immunity	0	0
$I_{i}^{A_{2}},I_{i}^{B_{2}}$	Secondarily infected hosts	0	0
*R*_*i*_	Recovery of hosts from secondary infection	0	0
*J*_*i*_	Juvenile mosquito population size	75,000,000	400,000
*Z*_*i*_	Total breeding mosquito population size	4,000,000	20,000
*M*_*i*_	Mature mosquito population size	3,996,000	20,000
$X_{i},Y_{i}$	Infected mosquitoes carrying serotype A, B	2000, 2000	0

The full list of state variables and their initial values for simulations. Suffix *i* is used to indicate parameters varying by patches (city and towns).

**Table 2:** Parameter table.

Symbol	Description	Default value	Refs.
*Dengue pathology parameters*			
*b*_1_	Conversion rate of susceptible hosts into infected	0.38	Alphey et al. (2011)
*b*_2_	Conversion rate of recovered hosts into secondarily infected	0.57	Alphey et al. (2011)
*α*_1_	Mortality rate of hosts with a primary infection	0.000457 per day	Atkinson et al. (2007)
*α*_2_	Mortality rate of hosts with a secondary infection	0.00833 per day	Guzman and Kouri (2002)
*ρ*_1_	Recovery rate of hosts from primary infection	0.17 per day	Alphey et al. (2011)
*ρ*_2_	Recovery rate of hosts from secondary infection	0.17 per day	Alphey et al. (2011)
*η*	Rate of loss of cross-immunity	0.00833 per day	Alphey et al. (2011)

*Aedes aegypti parameters*			
a	Biting rate per *Aedes aegypti* adult	0.5 per day	Alphey et al. (2011)
*g*	The number of eggs laid by adult mosquitoes	0.7 per day	Alphey et al. (2011)
*δ*	A density-dependent parameter	0.1	Bellows (1981)
*ϕ*	The rate at which juvenile larvae develop into mature adults	0.0541 per day	Alphey et al. (2011)
*μ*	Adult *Aedes* mortality rate	0.0741 per day	Alphey et al. (2011)
c	Conversion rate of mature mosquitoes into dengue vectors	0.38	Alphey et al. (2011)

*Network configuration parameters*			
*ω*_*ij*_	Commuter flow. Here a connectivity measure from town j to i	0 or 1	
*m*_*S*_	Proportion of susceptible hosts who commute	0.1	
*m*_*I*_	Proportion of infected hosts with a primary infection who commute	0.1	
$m_{I_{2}}$	Proportion of infected hosts with a secondary infection who commute	0.01	
*m*_*R*_	Proportion of recovered hosts who commute	0.1	

*Control parameters*			
*γ*_*i*_	Vaccination rate	$0<\gamma_{i}<1$	
*ζ*	(Im)perfect vaccine switch	0 or 1	
*Ψ*_*i*_	A number of released modified mosquitoes	Ratios X:1 to wild-type, where 0<X<10	
*ϵ*	Gene lethality	0<ϵ<1	

The full list of parameter terms and their default values for simulations. Suffix *i* is used to indicate parameters varying by patches (city and towns).

**Table 3:** R0 expressions based on different commuter behaviour. Movement influences the initial spread of an epidemic in a two patch network. The initial rate of spread is contingent on the ratio of vectors:hosts, with altered commuter flow adding to the complexity with which host population size is incorporated into R0.

Commute type	*ω*_*ij*_	*ω*_*ji*_	R0 expression
None	0	0	$\frac{a^{2}b_{1}{\mathrm{cM}}_{i}}{\mu (\alpha_{1}+\rho_{1})N_{i}}$
One way	1	0	$\frac{a^{2}b_{1}{\mathrm{cM}}_{i}N_{i}}{\mu (\alpha_{1}+\rho_{1}){\left(N_{i}+m_{S}N_{j}\right)}^{2}}$
Bidirectional	1	1	$\frac{a^{2}b_{1}{\mathrm{cM}}_{i}N_{i}{\left(1-m_{S}\right)}^{2}}{\mu (\alpha_{1}+\rho_{1}){\left(N_{i}+m_{S}(N_{j}-N_{i})\right)}^{2}}$

## Appendix A Supplementary data

Application 1
