# Supplementary material for: The interplay of vaccination and vector control on small dengue networks
Source: J Theor Biol. 2016 Oct 21;407:349–61. doi: 10.1016/j.jtbi.2016.07.034 (PMC5016021; doi:10.1016/j.jtbi.2016.07.034)
Supplement: Application 1 [file mmc1.pdf]

The transmission and control of dengue fever on small networks: Supplementary Information

1 Deriving the R0 equation

Here we present the full derivation for R0 in a two-patch network for a single serotype, as transmission occurs between vectors-hosts and hosts-vectors. We follow Diekmann et al., 2010, and divide the derivation into six stages for clarity.

1.1 Identify the infected subsystem

We begin by identifying which equations in the full set of ODEs describe new infections and changes in states of existing infections. As we are interested in the spread of a single serotype in this analysis, these equations are simplified by the elimination of secondary infection dynamics. Ω_i is the daily change in population size of patch i as a result of movement of individuals around the network. In a two patch network this must be equivalent to the loss of individuals from patch j, hence $\Omega_i = -\Omega_j$. Modifying equations (2) & (13) according to these criteria, (methods, section 2.4), we therefore have the change in infected hosts. I_i, and infected vectors X_i in patch i described by:

$$\frac{dI_i}{dt} = \frac{ab_1 X_i(1-\omega_{ji}m_S)S_i}{N_i+\Omega_i} + \frac{ab_1 X_j \omega_{ji} m_S S_i}{N_j - \Omega_i} - I_i(\alpha_1+\rho_1) \tag{1}$$

$$\frac{dX_i}{dt} = \frac{acM_i(1-\omega_{ji}m_I)I_i}{N_i+\Omega_i} + \frac{acM_i\omega_{ij}m_I I_j}{N_i+\Omega_i} - \mu X_i \tag{2}$$

Similarly we can model the dynamics of patch j infected hosts and vectors by:

$$\frac{dI_j}{dt} = \frac{ab_1X_j(1-\omega_{ij}m_S)S_j}{N_j-\Omega_i} + \frac{ab_1X_i\omega_{ij}m_SS_j}{N_i+\Omega_i} - I_j(\alpha_1+\rho_1) \tag{3}$$

$$\frac{dX_j}{dt} = \frac{acM_j(1-\omega_{ij}m_I)I_j}{N_j-\Omega_i} + \frac{acM_j\omega_{ji}m_II_i}{N_j-\Omega_i} - \mu X_j \tag{4}$$

1.2 Linearize subsystem about infection free steady state

Next we assume that at the absolute start of the epidemic, the change in state of susceptible hosts is negligible, $S_i = N_i$, $S_j = N_j$. Furthermore demographic changes to the adult mosquito population are also negligible, ${}^{dM_i}/_{dt} = 0$. As a result, equations (1)-(4) become functions only of other components included within the subsystem, with other state variables being reduced to constants. Hence,

$$\frac{dI_i}{dt} = \frac{ab_1(1-\omega_{ji}m_S)N_i}{N_i+\Omega_i}X_i + \frac{ab_1m_S\omega_{ji}N_i}{N_j-\Omega_i}X_j - (\alpha_1+\rho_1)I_i \tag{5}$$

$$\frac{dX_i}{dt} = \frac{acM_i(1-\omega_{ji}m_I)}{N_i+\Omega_i}I_i + \frac{acM_i\omega_{ij}m_I}{N_i+\Omega_i}I_j - \mu X_i \tag{6}$$

$$\frac{dI_j}{dt} = \frac{ab_1(1-\omega_{ij}m_S)N_j}{N_j-\Omega_i}X_j + \frac{ab_1\omega_{ij}m_SN_j}{N_i+\Omega_i}X_i - (\alpha_1+\rho_1)I_j \tag{7}$$

$$\frac{dX_j}{dt} = \frac{acM_j(1-\omega_{ji}m_I)}{N_j-\Omega_i}I_j + \frac{acM_j\omega_{ji}m_I}{N_j-\Omega_i}I_i - \mu X_j \tag{8}$$

1.3 Subsystem decomposition

Each equation in the subsystem has three components. The first corresponds to the rate of infections occuring within the focal patch, the second term models infections that are facilitated by host movement, and the final term accounts for mortality and recovery from infections. To keep track of these components in an easy manner, new notation is introduced here, where rate $\dot{I_{\gamma\delta}}$ corresponds to transmission events depending on patch δ that contribute to infections in γ. With this notation is becomes straightforward to decompose the infection subsystem into transmission rates and transitions rates to form the next generation matrix. For example, equation (5) can be rewritten as:

$$\frac{dI_i}{dt} = \dot{I_{ii}} + \dot{I_{ij}} - (\alpha_1 + \rho_1)I_i \tag{9}$$

Next we set $y = (I_i, I_j, X_i, X_j)'$, where $'$ indicates the transpose of this vector. Now we write the linearized infection subsystem in the form:

$$\dot{y} = (\mathbf{T} + \mathbf{\Sigma})y \tag{10}$$

Where $\mathbf{T}$ corresponds to transmissions, covering all epidemiological events giving rise to new infections, whilst $\mathbf{\Sigma}$ corresponds to all transitions, which encompasses all other rates responsible for changes in numbers of infections, namely, mortality and recovery rates. Infected states are referred to with indices α and β, with $\alpha, \beta \in 1, 2, 3, 4$, such that $\mathbf{T}_{\alpha\beta}$ represents the rate at which individuals in state β give rise to individuals in infected state α. Hence,

$$\mathbf{T} = \begin{array}{c} \\ I_i \\ I_j \\ X_i \\ X_j \end{array} \begin{array}{c} \begin{array}{cccc} I_i & I_j & X_i & X_j \end{array} \\ \begin{pmatrix} 0 & 0 & \dot{I_{ii}} & \dot{I_{ij}} \\ 0 & 0 & \dot{I_{ji}} & \dot{I_{jj}} \\ \dot{X_{ii}} & \dot{X_{ij}} & 0 & 0 \\ \dot{X_{ji}} & \dot{X_{jj}} & 0 & 0 \end{pmatrix} \end{array} . \tag{11}$$

Whilst

$$\mathbf{\Sigma} = \begin{array}{c} \\ I_i \\ I_j \\ X_i \\ X_j \end{array} \begin{array}{c} \begin{array}{cccc} I_i & I_j & X_i & X_j \end{array} \\ \begin{pmatrix} -(\alpha_1 + \rho_1) & 0 & 0 & 0 \\ 0 & -(\alpha_1 + \rho_1) & 0 & 0 \\ 0 & 0 & -\mu & 0 \\ 0 & 0 & 0 & -\mu \end{pmatrix} \end{array} . \tag{12}$$

1.4 Calculate Next Generation Matrix

The Next Generation Matrix (NGM), $\mathbf{K}$, is defined as $\mathbf{K} = -\mathbf{T\Sigma}^{-1}$. The biological interpretation for this is clear, because $-\mathbf{\Sigma}^{-1}_{\alpha\beta}$ is the expected time that an individual in state β will spend in state α during their epidemiological life. Since $\mathbf{\Sigma}$ forms a diagonal matrix here, it is intuitive that this life span will equal $\frac{1}{rate\ of\ recovery\ or\ death}$. Hence,

$$\mathbf{K} = \begin{pmatrix} 0 & 0 & \dot{I_{ii}} & \dot{I_{ij}} \\ 0 & 0 & \dot{I_{ji}} & \dot{I_{jj}} \\ \dot{X_{ii}} & \dot{X_{ij}} & 0 & 0 \\ \dot{X_{ji}} & \dot{X_{jj}} & 0 & 0 \end{pmatrix} \times \begin{pmatrix} \frac{1}{(\alpha_1+\rho_1)} & 0 & 0 & 0 \\ 0 & \frac{1}{(\alpha_1+\rho_1)} & 0 & 0 \\ 0 & 0 & \frac{1}{\mu} & 0 \\ 0 & 0 & 0 & \frac{1}{\mu} \end{pmatrix} \tag{13}$$

$$= \begin{pmatrix} 0 & 0 & \frac{\dot{I_{ii}}}{\mu} & \frac{\dot{I_{ij}}}{\mu} \\ 0 & 0 & \frac{\dot{I_{ji}}}{\mu} & \frac{\dot{I_{jj}}}{\mu} \\ \frac{\dot{X_{ii}}}{\alpha_1+\rho_1} & \frac{\dot{X_{ij}}}{\alpha_1+\rho_1} & 0 & 0 \\ \frac{\dot{X_{ji}}}{\alpha_1+\rho_1} & \frac{\dot{X_{jj}}}{\alpha_1+\rho_1} & 0 & 0 \end{pmatrix}. \tag{14}$$

1.5 Split the Next Generation Matrix into components

Next the NGM is split into seperate matrices for rates of vector-to-host and host-to-vector transmission, represented by $\mathbf{K_{V\to H}}$ and $\mathbf{K_{H\to V}}$. Hence,

$$\mathbf{K_{V\to H}} = \frac{1}{\mu} \begin{pmatrix} \dot{I_{ii}} & \dot{I_{ij}} \\ \dot{I_{ji}} & \dot{I_{jj}} \end{pmatrix}. \tag{15}$$

$$\mathbf{K_{H\to V}} = \frac{1}{\alpha_1+\rho_1} \begin{pmatrix} \dot{X_{ii}} & \dot{X_{ij}} \\ \dot{X_{ji}} & \dot{X_{jj}} \end{pmatrix}. \tag{16}$$

From this arrangement two R0s can be derived: $R0_{V\to H}$ and $R0_{H\to V}$, by finding the dominant eigenvalue of matrices (15) & (16).

1.6 Calculating the dominant eigenvalue

The determinant is taken of the vector-to-host matrix minus the eigenvalue, λ, multiplied by identity matrix $\mathbf{N}$.

$$\left|\mathbf{K}_{\mathbf{V}\to\mathbf{H}} - \lambda\mathbf{N}\right| = \frac{1}{\mu}\begin{vmatrix} \dot{I}_{ii} - \mu\lambda & \dot{I}_{ij} \\ \dot{I}_{ji} & \dot{I}_{jj} - \mu\lambda \end{vmatrix} \tag{17}$$

$$= \frac{1}{\mu}\ [\ (\dot{I}_{ii} - \mu\lambda)(\dot{I}_{jj} - \mu\lambda) - \dot{I}_{ij}\dot{I}_{ji}\ ] \tag{18}$$

$$= \frac{1}{\mu}\ [\ \mu^2\lambda^2 - \mu(\dot{I}_{ii} + \dot{I}_{jj})\lambda + \dot{I}_{ii}\dot{I}_{jj} - \dot{I}_{ij}\dot{I}_{ji}\ ] \tag{19}$$

$$= \lambda^2 - \frac{\dot{I}_{ii} + \dot{I}_{jj}}{\mu}\lambda + \frac{\dot{I}_{ii}\dot{I}_{jj} - \dot{I}_{ij}\dot{I}_{ji}}{\mu^2} \tag{20}$$

$$= 0\ . \tag{21}$$

The absolute largest solution to this quadratic equation gives the dominant eigenvalue, hence:

$$\left(\lambda - \frac{\dot{I}_{ii} + \dot{I}_{jj}}{2\mu}\right)^2 - \left(\frac{\dot{I}_{ii} + \dot{I}_{jj}}{2\mu}\right)^2 + \frac{\dot{I}_{ii}\dot{I}_{jj} - \dot{I}_{ij}\dot{I}_{ji}}{\mu^2} = 0 \tag{22}$$

By rearrangement an expression for λ can be found:

$$\left(\lambda - \frac{\dot{I_{ii}} + \dot{I_{jj}}}{2\mu}\right)^2 = \left(\frac{\dot{I_{ii}} + \dot{I_{jj}}}{2\mu}\right)^2 - \frac{(\dot{I_{ii}}\dot{I_{jj}} - \dot{I_{ij}}\dot{I_{ji}})}{\mu^2} \tag{23}$$

$$\left(\lambda - \frac{\dot{I_{ii}} + \dot{I_{jj}}}{2\mu}\right) = \left|\sqrt{\frac{(\dot{I_{ii}} + \dot{I_{jj}})^2}{(2\mu)^2} - \frac{(\dot{I_{ii}}\dot{I_{jj}} - \dot{I_{ij}}\dot{I_{ji}})}{\mu^2}}\right| \tag{24}$$

$$\lambda = \frac{\dot{I_{ii}} + \dot{I_{jj}}}{2\mu} + \left|\sqrt{\frac{(\dot{I_{ii}} + \dot{I_{jj}})^2}{(2\mu)^2} - \frac{4(\dot{I_{ii}}\dot{I_{jj}} - \dot{I_{ij}}\dot{I_{ji}})}{(2\mu)^2}}\right| \tag{25}$$

$$= \frac{\dot{I_{ii}} + \dot{I_{jj}}}{2\mu} + \frac{\left|\sqrt{(\dot{I_{ii}} + \dot{I_{jj}})^2 - 4(\dot{I_{ii}}\dot{I_{jj}} - \dot{I_{ij}}\dot{I_{ji}})}\right|}{\left|\sqrt{(2\mu)^2}\right|} \tag{26}$$

$$= \frac{1}{2\mu}\left(\dot{I_{ii}} + \dot{I_{jj}} + \left|\sqrt{(\dot{I_{ii}} + \dot{I_{jj}})^2 - 4(\dot{I_{ii}}\dot{I_{jj}} - \dot{I_{ij}}\dot{I_{ji}})}\right|\right) \tag{27}$$

$$= \frac{1}{2\mu}\left(\dot{I_{ii}} + \dot{I_{jj}} + \left|\sqrt{\dot{I_{ii}}^2 + \dot{I_{jj}}^2 + 2\dot{I_{ii}}\dot{I_{jj}} - 4\dot{I_{ii}}\dot{I_{jj}} + 4\dot{I_{ij}}\dot{I_{ji}}}\right|\right) \tag{28}$$

$$\therefore \lambda = \frac{1}{2\mu}\left(\dot{I_{ii}} + \dot{I_{jj}} + \left|\sqrt{(\dot{I_{ii}} - \dot{I_{jj}})^2 + 4\dot{I_{ij}}\dot{I_{ji}}}\right|\right) \tag{29}$$

Given the algebraic similarity between equations (15) & (16), for $\mathbf{K_{V\to H}}$ and $\mathbf{K_{H\to V}}$ respectively, it follows that by the preceeding derivation:

$$R0_{V\to H} = \frac{1}{2\mu}\left(\dot{I_{ii}} + \dot{I_{jj}} + \left|\sqrt{(\dot{I_{ii}} - \dot{I_{jj}})^2 + 4\dot{I_{ij}}\dot{I_{ji}}}\right|\right) \tag{30}$$

$$R0_{H\to V} = \frac{1}{2(\alpha_1 + \rho_1)}\left(\dot{X_{ii}} + \dot{X_{jj}} + \left|\sqrt{(\dot{X_{ii}} - \dot{X_{jj}})^2 + 4\dot{X_{ij}}\dot{X_{ji}}}\right|\right) \tag{31}$$

These equations form the basis of the R0 analysis given in the main text.

2 Exploring Network Asymmetry

Assumptions about host movement were analysed by taking different commuter behaviours into account, as demonstrated in figure 1.

Figure 1: Four satellite, wheel-spoke design network configurations. Central city patch i connects to every other patch, but smaller town patches can only commute through the city. Schematic (a) displays the one-way commute type, where daily commuters come from the towns to the city and back again. Alternatively, (b) shows the bidirectional commuter configuration, where in addition to the one-way model, city inhabitants may also commute to the towns.

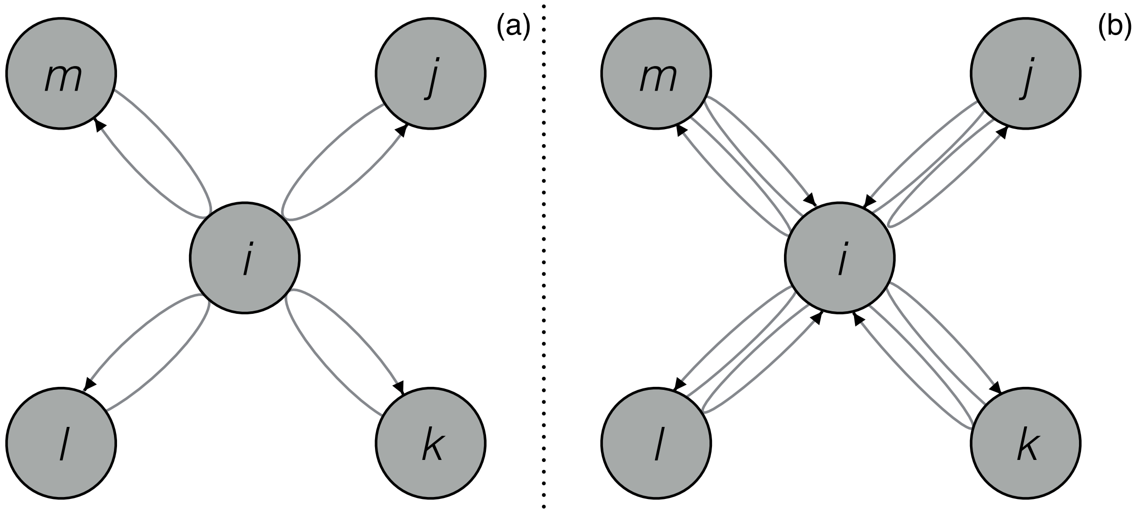

'City-only' and 'town-only' control strategies were also compared when commuter flow between patches varied. So at a given level of control (2:1 insect release and 1% vaccination coverage) the effectiveness of controls on one way commute network types were compared to those generated when bidirectional commuting is permitted, with a commuter flow of 0.1 (figure 2).

As the one way plot (a) indicates, town-only control is more effective than city-only, with the difference increasing as more satellite patches are added to the network. This increase makes sense, as town-only control covers a larger proportion of the susceptible population for every additional town in the network. However, once bidirectional commuting is permitted, (b), this result is not so clear cut. Small networks benefit more from city-only controls under these conditions. The town-only method still becomes more effective as network size increases, but this method is less effective for bidirectional movement on networks.

One possible explanation for this is that bidirectional commuting reduces the city population size and increases the size of the towns relative to the one-way commute network. As a result, the ratio of vectors:hosts is higher in cities, biasing biting events in the city. Hence most infections in the bidirectional network are not occurring in towns, but the city instead, and control there can play a larger role in reducing infections across the network, particularly in smaller networks. This does not hold for the four satellite network under town-only control. It is likely that the larger pool of town-dwelling susceptible individuals in larger networks may benefit even more from town-only controls, which offsets the high probability of individuals being bitten in the city.

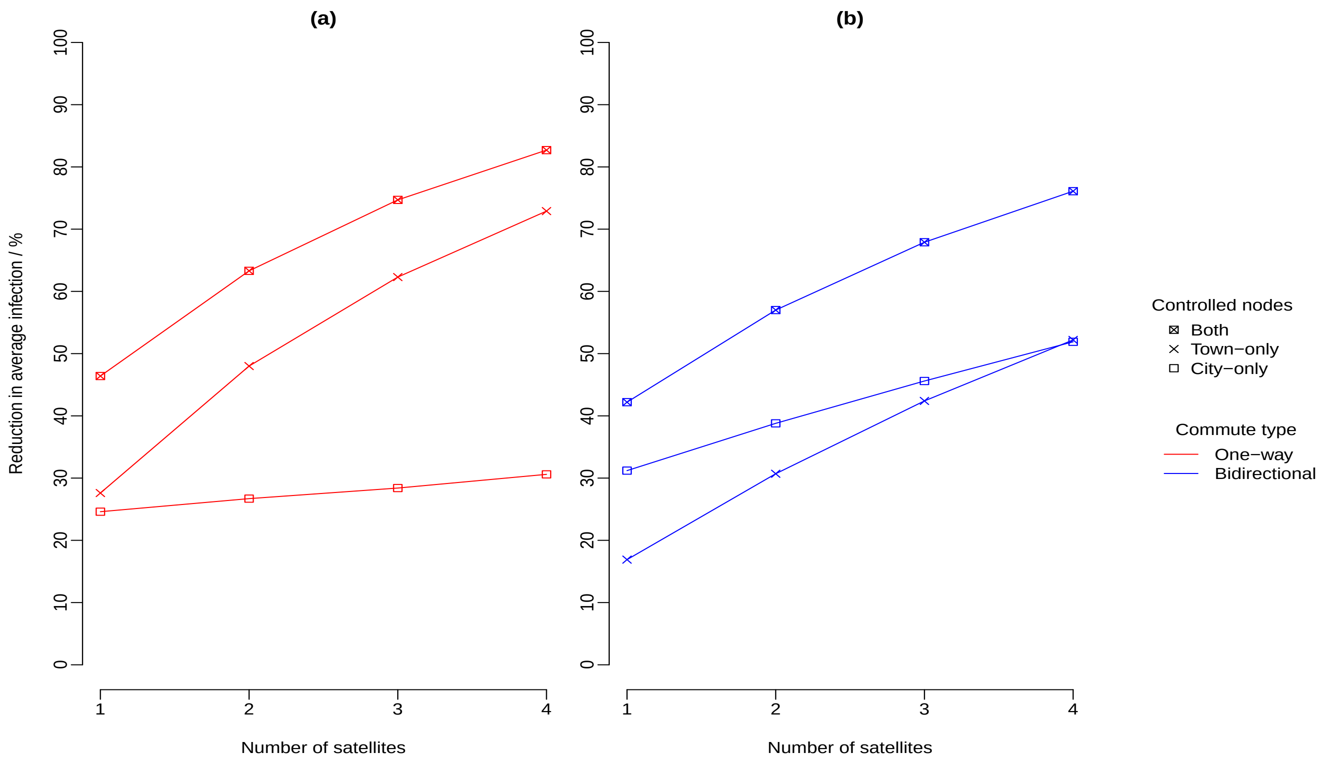

Figure 2: The effectiveness of dengue fever control, as measured by the percentage reduction in cases observed when control is applied, differs by commuter flow dynamics. For one-way commutes, (a), town-only control leads to a greater reduction in infections than city-only. The reverse is observed for bidirectional commutes, (b), though only for very small networks.

This city-biased biting effect is seen when just looking at DHF cases as well. Concordantly over the range of network sizes explored, city-only control is the most effective method for controlling these severe cases.

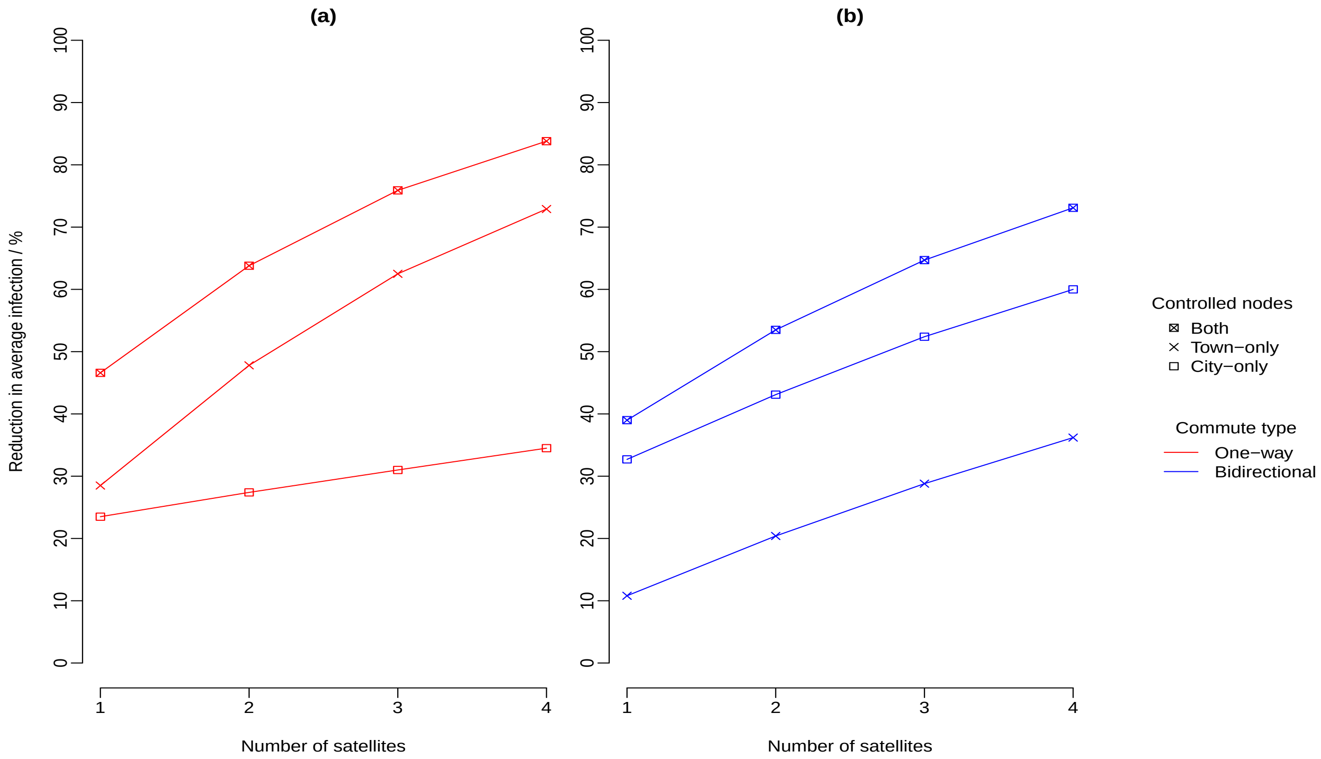

Figure 3: Secondary dengue control effectiveness differs by commute flow, with (a) displaying simulation results for a network using only one-way commuter behaviour, whilst (b) was generated using networks permitting bidirectional movements. All strategies become more effective as the networks increase in size, but the most effective strategy differs between (a) and (b).

From figure 3 it seems that commuter flow heavily influences where control measures will be most effective. In this case, more symmetry in commutes among patches brought about by bidirectional commutes makes biting events more common in the city, hence secondary infections disproportionately arise here, reducing the ability of town-only controls to target these cases.

Here we can also see that for a four satellite network under bidirectional commuting, town-only control is more effective at reducing primary infections, but city-only control is more effective at reducing secondary cases. This dichotomy in efficacy of control measures creates a trade-off

when considering management of large networks. On the one hand, town-only control deals with a large number of mildly ill individuals, but city-only control will prevent a small number of people from developing very serious clinical syndromes.

3 Sensitivity Analysis

The model was examined using sensitivity analysis, where all aspects of the model were kept the same but one parameter was varied at a time. Vector parameters were increased by 5, 15, and 25%, and the subsequent increase in primary infections, as compared to a simulation where no parameters were augmented, was plotted. The results displayed in figure 4 show which parameters have most influence in the network.

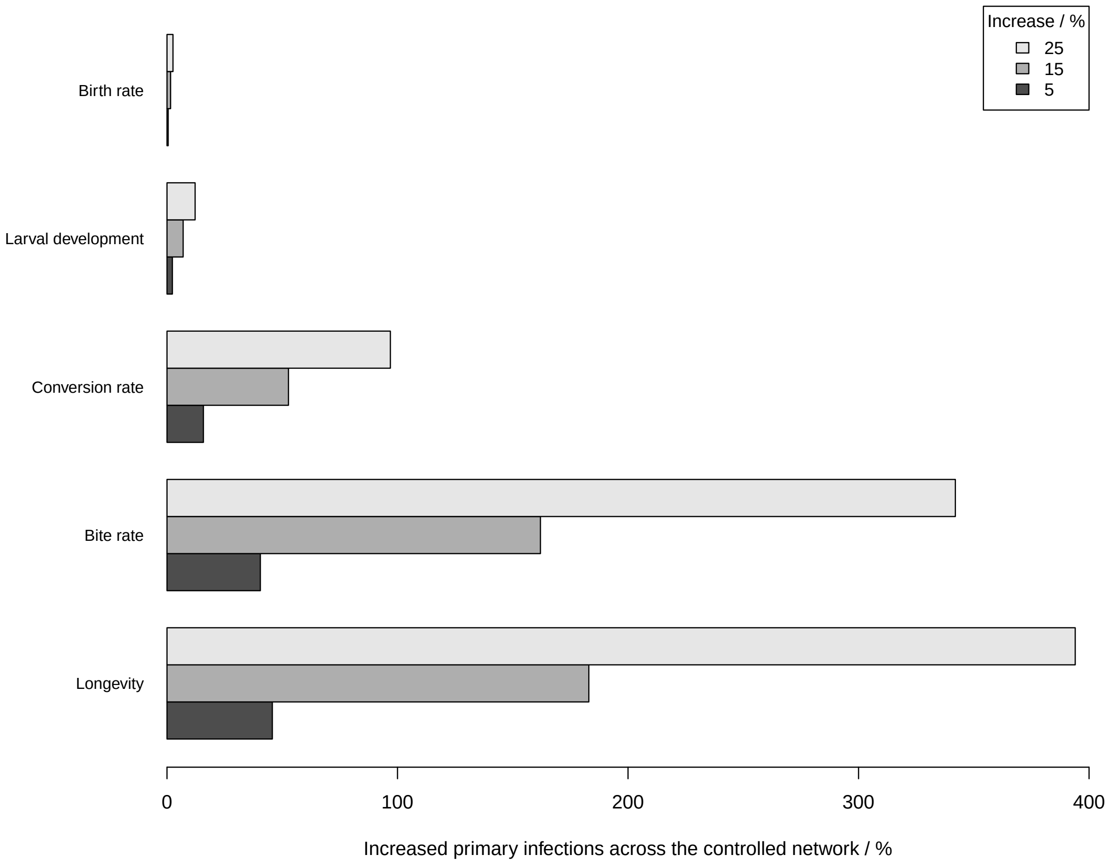

Figure 4: Vector parameter sensitivity. Increasing the value of these vector parameters increases the number of infections that occur. Birth rate, larval development and vector longevity, the inverse of mortality rate, determine population dynamics of the vector, whilst bite rate and conversion rate of mature insects into dengue-carrying vectors determine disease transmissability.

As is apparent, there is a range of responses that the system undergoes in response to altering different variables. Hence in trying to determine context appropriate control measures, mosquito

biology is crucially important. This also indicates how important it is to gain accurate measures of mosquito biology in the field if accurate predictions are to be made by the model. For example, a 5% error in bite rate can lead to a 45% increase in the expected mean number of infected individuals.

Once again these effects are further amplified for secondary cases, as indicated by figure 5. In addition, for secondary dengue, bite rate replaces longevity as the parameter to which the model is most sensitive. This is likely due to the increased dependence on multiple biting events per individual that are required to propagate these cases, relative to primary cases.

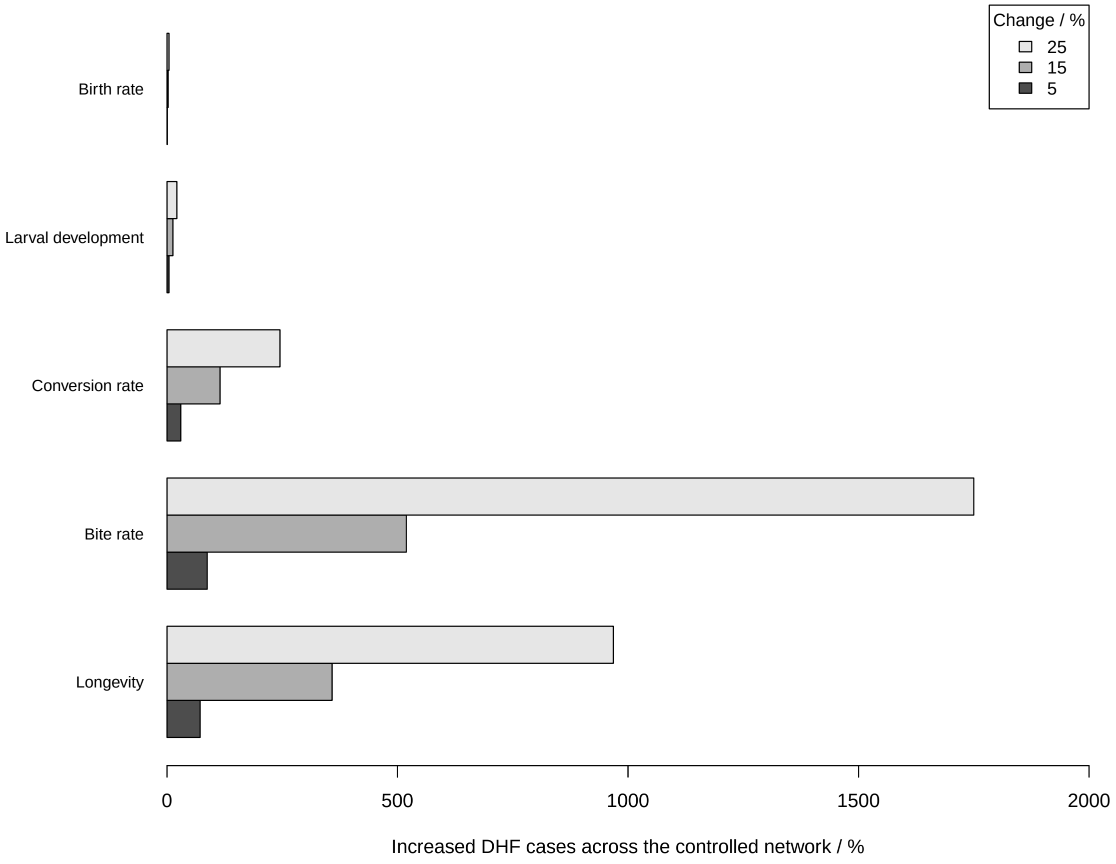

Figure 5: Dramatic increases to secondary dengue in vector parameter stressed networks. As for figure 14, birth rate, larval development and vector longevity determine population dynamics of the vector, whilst bite rate and conversion rate of mature insects into dengue-carrying vectors determine disease transmissability.

Given how important commuter flow seems to be in altering the outcome of dengue fever epidemics and indeed the effectiveness of controls, sensitivity analysis was also applied to this (figure 6).

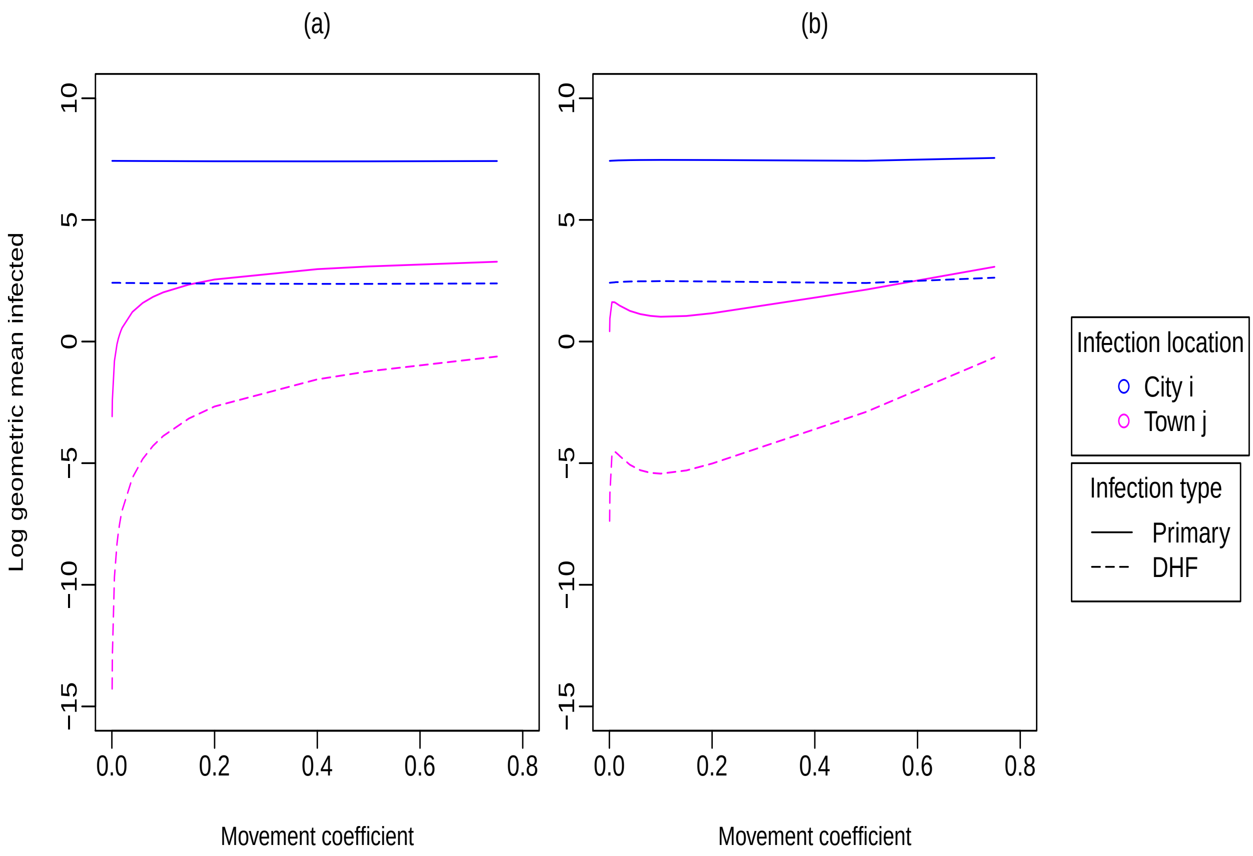

Figure 6: Breakdown of infection locations for one-way (a) and bidirectional (b) network types on a logarithmic scale. The movement coefficient is calculated as the product of connectivity and class movement terms, hence indicating the proportion of each patch that commutes. City patch infections are relatively resilient to changes in movement coefficient, whilst town infections are more sensitive to differences both in the magnitude and direction of commutes.

The city patch is relatively unaffected by the increase in commute traffic, but the level of infection in town patches varies considerably. For the one way commute type, (a), increased commuter flow around the network spreads the infection around the network leading to a greater number of infections in the towns. However this response becomes limited as the epidemic becomes constrained by other epidemiological factors (e.g. conversion rate of susceptible hosts to infected) more so than movement.

To an extent bidirectional movement shows a similar pattern, (b), however there is a difference between this line and the one-way network for intermediate commute values. This difference may be explained by fluctuating strength of town vector:host ratios in the bidirectional network, which varies as a function of the movement coefficient. This is also in keeping with the predictions made from R0 calculations, main text section 3, providing yet more evidence for the important role of commuting behaviour in altering the outcome of dengue outbreaks.

In terms of commute directionality, bidirectionality, with a commuter flow of 0.1, may actually mean that an unrealistic number of people enter a town daily from the city (in this particular example, town sizes fluctuate by a factor of five on a daily basis). So it may be that this effect may not occur so strongly in real systems. However, it is useful to illustrate that commuter dynamics may actually fall somewhere between one-way and bidirectional types explored here. Hence bidirectional and one-way network results could be viewed as broad limits for describing how movement out of large, well-connected patches may influence epidemic dynamics.
